# Efficacy and Safety of Palivizumab as a Prophylaxis for Respiratory Syncytial Virus (RSV) Disease: An Updated Systemic Review and Meta-Analysis

**DOI:** 10.7759/cureus.51375

**Published:** 2023-12-31

**Authors:** Khaled El-Atawi, Daniele De Luca, Ranagasamy Ramanathan, Manuel Sanchez Luna, Saad Alsaedi, Muzafar Gani Abdul Wahab, Moataz Hamdi, Maysa Saleh

**Affiliations:** 1 Department of Pediatrics, Latifa Women and Children Hospital, Dubai, ARE; 2 Division of Pediatrics and Neonatal Critical Care, "Antoine Béclère" Hospital, Paris Saclay University Hospitals, APHP (Assistance Publique Hôpitaux de Paris), Paris, FRA; 3 Physiopathology and Therapeutic Innovation Unit, INSERM (Institut National de la Santé Et de la Recherche Médicale) U999, Paris Saclay University, Paris, FRA; 4 Division of Neonatology, Department of Pediatrics, Los Angeles General Medical Center, Keck School of Medicine of USC (University of Southern California), Los Angeles, USA; 5 Department of Neonatology, University Hospital Gregorio Marañón, Madrid, ESP; 6 Department of Pediatrics and Neonatology, King Faisal Specialist Hospital & Research Centre, Jeddah, SAU; 7 Department of Pediatrics and Neonatology, McMaster University, Hamilton, CAN; 8 Department of Pediatrics, Al Jalila Children's Speciality Hospital, Dubai, ARE; 9 Department of Pediatrics and Child Health, Al Jalila Children's Speciality Hospital, Dubai, ARE

**Keywords:** monoclonal antibodies, chronic lung disease, lower respiratory tract infection, palivizumab, respiratory syncytial virus (rsv)

## Abstract

This systematic review and meta-analysis aimed to summarize the current evidence regarding the efficacy and safety of palivizumab as a prophylaxis for respiratory syncytial virus (RSV) disease. We searched MEDLINE via PubMed, Scopus, Cochrane, Web of Science, Embase, and Science Direct from inception till November 2023. Studies that assessed the efficacy and safety of palivizumab in infants aged between 28 days and three months of age were included. We analyzed the data using Review Manager 5.4 software, with results pooled across studies and expressed as risk ratios (RR) with 95% confidence intervals (CI). A total of 10 studies were included. The effect estimates favored palivizumab over placebo regarding the hospitalization for RSV infection (RR=0.51, 95% CI: 0.40 to 0.65; P<0.00001) and ICU admission (RR=0.49, 95% CI: 0.30 to 0.81; P=0.005). On the other hand, the effect estimate showed no significant difference between palivizumab and placebo regarding all-cause mortality (RR=0.69, 95% CI: 0.42 to 1.15; P=0.16), lower respiratory tract infection (RR=0.42, 95% CI: 0.11 to 1.69; P=0.22), and need for mechanical ventilation (RR=0.75, 95% CI: 0.34 to 1.67; P=0.48). Palivizumab can be considered a prophylaxis for RSV disease in young children as it is safe, well-tolerated, and effective in reducing RSV hospitalizations. However, further research through high-quality randomized controlled trials is required to determine its efficacy as a therapeutic agent for established RSV infections.

## Introduction and background

The respiratory syncytial virus (RSV) is a leading cause of acute upper and lower respiratory tract infection in all ages, but it is most common in the elderly, critically immunocompromised individuals, and the first year of life children [1]. During the winter, infection rates rise to reach their highest rate, known as "RSV seasons" [2]. It was reported that RSV causes significant morbidity in children in the form of many respiratory problems, including high wheezing rates and allergies [3]. In addition, the presence of pre-existing disorders increases the risk of death from severe RSV infection [4]. RSV mortality was 8.6% in a hospital-based cohort study in the United Kingdom (UK), with a standardized mortality ratio of 0.76, and all RSV deaths were linked to pre-existing disorders, particularly cardiac abnormalities and several co-morbidities [5]. RSV was also found to be a significant infection in patients who had solid organ transplants [6].

Palivizumab, a monoclonal antibody targeting the RSV F glycoprotein, is being considered as a potential prophylactic for severe RSV infection. It has demonstrated effectiveness in reducing RSV-related hospitalizations in infants with chronic lung disease, congenital heart disease, and those born prematurely before 36 weeks of gestation [7]. Palivizumab is currently widely used as a prophylaxis in these high-risk categories in several countries. Infants can utilize it with very few incidences of anaphylaxis [8]. RSV immune globulin is the precursor of palivizumab for RSV prophylaxis. Many studies have reported that this drug does not reduce the length of hospitalization or intensive care unit stays in high-risk or previously healthy children when administered therapeutically [9]. Despite this, palivizumab is sometimes prescribed to individuals with severe RSV disease or those who are at high risk of developing an RSV infection. Despite the widespread use of palivizumab as RSV prophylaxis, several concerns exist regarding its efficacy and safety as a therapeutic agent for treating active RSV infections. Palivizumab provides passive immunity against RSV, but multiple studies have found no significant benefits of palivizumab therapy in reducing the length of hospital stay, ICU admission, or the need for mechanical ventilation in children hospitalized with RSV [10-12]. Treatment with palivizumab after the onset of RSV illness appears to have minimal impact on these indicators of disease severity. In addition, the high costs of palivizumab limit its cost-effectiveness as a therapeutic agent. The average cost per patient for palivizumab treatment ranges from $4000-$8000, leading to debates about its value relative to potential benefits [13].

This systematic review and meta-analysis summarizes the current evidence on the prophylactic use of palivizumab for RSV infections in infants and children regarding key outcomes such as mortality, disease severity, and hospital length of stay. We aimed to guide evidence-based recommendations on the role of palivizumab as a prophylactic option for severe RSV disease.

## Review

Material and methods

We followed the Preferred Reporting Items for Systematic Reviews and Meta-Analyses (PRISMA) statement guidelines [14] during this systematic review and meta-analysis preparation and performed all steps according to the Cochrane Handbook of Systematic Reviews of Intervention [15].

Search Strategy

We searched MEDLINE via PubMed, Scopus, Cochrane, Web of Science, Embase, and Science Direct from inception till November 2023 using relevant keywords. We used the following search strategy for searching different databases: (Palivizumab OR "MEDI 493" OR Monoclonal Antibody MEDI-493 OR "Monoclonal Antibody MEDI 493" OR Monoclonal Antibody MEDI493 OR "MEDI-493" OR "MEDI493" OR Synagis) AND ("Respiratory Syncytial Virus" OR "Syncytial Virus, Respiratory" OR "Syncytial Viruses, Respiratory" OR "Virus, Respiratory Syncytial" OR "Viruses, Respiratory Syncytial" OR "Chimpanzee Coryza Agent" OR "Chimpanzee Coryza Agents" OR "Coryza Agent, Chimpanzee" OR "Coryza Agents, Chimpanzee").

Eligibility Criteria and Study Selection

We included studies that followed these criteria: (1) Infants between 28 days and three months of age, (2) designed as randomized controlled trials (RCTs), cohort or case controls either prospective or retrospective, (3) written in English, and (4) reporting any outcome. We excluded conference abstracts or unpublished data, studies written in a language other than English, in-vitro studies, and duplicated articles by the same author unless those with longer follow-up studies. Two independent reviewers (K.E. and R.R.) performed the title and abstract screening. Any disagreements between the two reviewers at this stage were resolved through discussion, or, if necessary, a third reviewer (M.S.) was consulted. Studies that appeared to meet the inclusion criteria, or for which there was insufficient information in the title and abstract to make a clear decision, were advanced to full-text review. Again, two independent reviewers (M.S.L. and M.E.) assessed each full-text article to determine its eligibility. Disagreements at this stage were resolved through consultation with a third reviewer (M.S). The reference lists of all included studies were scanned to identify additional studies that might have been missed during the initial database searches. Any potentially relevant studies identified through this process were subjected to full-text review and included if they met the criteria.

Study Outcomes

We evaluate the efficacy and safety of palivizumab as an immunoprophylactic regarding hospitalization for RSV infection, admission to ICU, mechanical ventilation for RSV infection, number of children reporting related adverse events, All-cause mortality, and lower respiratory tract infection.

Quality Assessment

The risk of bias was evaluated by the ROB tool [16], which included the following risks: selection bias "through random sequence generation and allocation concealment," selective reporting, attrition bias, performance bias through blinding of participants and personnel, and detection bias through blinding of outcome assessment. Each bias domain is recorded as one of the following: low risk, high risk, or unclear risk. Also, the prospective or retrospective cohort was evaluated by quality assessment of cohort studies by NIH tool data extraction [17].

Data Extraction

For studies that met the inclusion criteria, relevant data were extracted by two reviewers independently (K.E and M.S) using a standardized data extraction form. This form was piloted on a subset of included studies and refined as needed. We obtained data from text, tables, figures (using Graph Grabber version 2.0), and supplementary data. All extracted data were further reviewed by a third reviewer (S.A). We extracted data regarding the study characteristics (study ID, duration, sample size, inclusion criteria, and conclusion) and outcomes.

Statistical Analysis

We conducted this meta-analysis by using Review Manager, Version 5.4 (Copenhagen: The Cochrane Collaboration, 2014). Regarding the study outcomes, risk ratio (RR) with 95% confidence interval (CI) was used for dichotomous variables, while the mean difference (MD) and 95% CI were presented for continuous variables. Cochrane's P values and I2 were tested to examine heterogeneity among the studies. High heterogeneity likely existed due to clinical and methodological factors, so the random effect model was adopted in this meta-analysis even though I2 was small. Funnel plots and the Egger regression test could not be performed due to the limited number of included trials (less than ten studies).

Results

**Figure 1:**
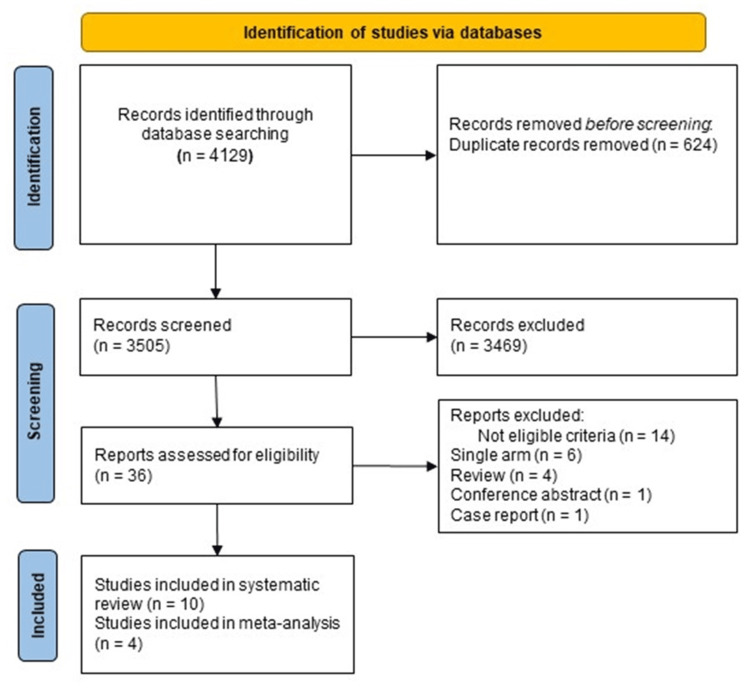
PRISMA flow diagram of the literature search results Preferred Reporting Items for Systematic Reviews and Meta-Analyses

The initial search resulted in 4129 articles. Fifty-four studies were retrieved from the manual search. Of these 4129 articles, we excluded 624 articles due to duplication. A total of 3505 articles underwent title and abstract screening, and 3469 were excluded because they did not meet the inclusion criteria. The remaining 36 articles underwent full-text screening. A total of 10 studies were finally included in the final qualitative synthesis; of them, only four studies were included in the quantitative analysis, as shown in Figure 1.

Risk of Bias Assessment of the Included Studies

**Figure 2:**
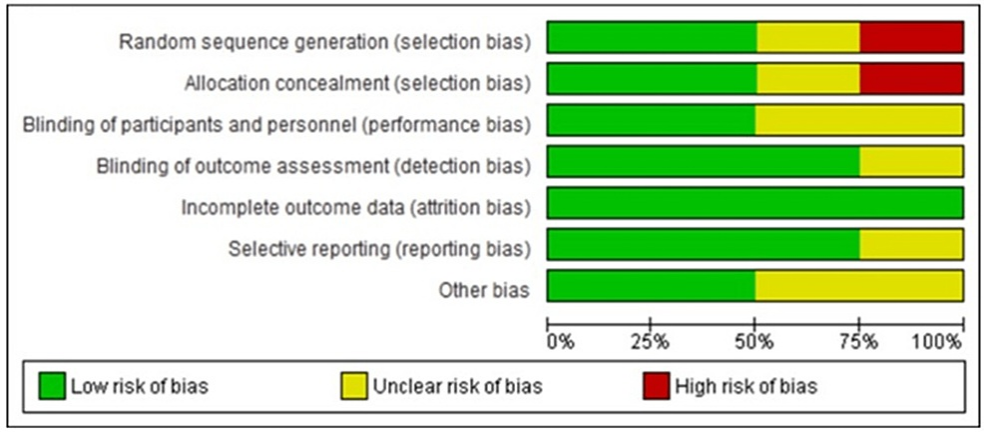
Risk of bias summary of the included studies

Feltes et al. (2003), rated a low risk of bias across all domains except for unclear risk for selective reporting and other biases [18]. The IMpact-RSV Study Group (1998) also had a low risk of bias for most domains, with unclear risk for performance and detection bias [19]. McCormick and Southern (2007) had a high risk of selection bias and an unclear risk for other biases [20]. Subramanian et al. (1998) had an unclear risk of bias in the selection and performance bias but low risk for the remaining domains [21]. The included cohort studies were judged as having fair quality based on the ROB tool and NIH assessment tool for risk of bias, as shown in Figure 2 and Table 1.

**Table 1:** Quality assessment of cohort studies by an NIH tool NA: not applicable, CD: cannot be determined, NR: not reported, NIH: National Institutes of Health

Domains	Giebels et al. (2008) [22]	Simoes et al. (2007) [23]	Simões et al. (2010) [24]	McCormick & Southern (2007) [20]	Yeo et al. (2021) [25]	Viguria et al. 2021 [26]
Item 1	Yes	Yes	Yes	Yes	Yes	Yes
Item 2	Yes	Yes	Yes	Yes	Yes	Yes
Item 3	Yes	Yes	Yes	Yes	Yes	Yes
Item 4	Yes	Yes	Yes	Yes	Yes	Yes
Item 5	NR	NR	Yes	NR	Yes	NR
Item 6	NA	NA	NA	NA	NA	NA
Item 7	NR	Yes	NR	Yes	Yes	NR
Item 8	NA	Yes	NA	NA	NA	NA
Item 9	NR	Yes	NR	Yes	Yes	Yes
Item 10	NA	NA	NA	NA	NA	NA
Item 11	Yes	Yes	Yes	Yes	Yes	Yes
Item 12	Yes	Yes	Yes	Yes	Yes	Yes
Item 13	Yes	Yes	Yes	Yes	Yes	Yes
Item 14	NA	NA	NA	NA	NA	NA
Total scores	9	9	9	9	10	8
Quality rating:	Fair quality	Fair quality	Fair quality	Fair quality	Fair quality	Fair quality

Characteristics of the Included Studies

The included studies range from randomized controlled trials to retrospective and prospective cohort studies conducted between 1998 and 2021. The sample sizes range from 42 to 1502 participants. The main outcomes examined are hospitalization rates, lower respiratory tract infections, ICU admission, adverse events, and effects on subsequent wheezing (Table 2).

**Table 2:** Study characteristics RCT: randomized controlled trials, ICU: intensive care unit

Study ID	Year	Study design	Sample size	Study's arms		Outcomes
Viguria et al. [26]	2021	Prospective cohort study	142	Palivizumab	No palivizumab	Hospitalized patients
Yei et al. [25]	2021	Retrospective cohort study	415	Palivizumab	No palivizumab	Variables associated with respiratory syncytial virus (RSV)
Simões et al. [24]	2010	Prospective cohort study	191	Palivizumab	Non-RSV hospitalized	lower respiratory tract infections, risk of hospitalization, and ICU admission rate
Giebels et al. [22]	2008	Retrospective cohort study	75	Palivizumab	No palivizumab	Hospital stay per patient and the incidence of milder forms of respiratory illness that did not require hospitalization
McCormick & Southern [20]	2007	National survey study	143	Palivizumab	Placebo	Lower respiratory tract infection and other related adverse events
Simoes et al. [23]	2007	Prospective cohort study	191	Palivizumab	No palivizumab	Lower respiratory tract infection and an atopic diathesis on subsequent recurrent wheezing
Cohen et al. [27]	2005	Phase 2 clinical trial	186	Palivizumab	Placebo	Lower respiratory tract infection and other related adverse events.
Feltes et al. [18]	2003	RCT	1287	15 mg/kg palivizumab (IM)	Placebo	Days of RSV hospitalization, RSV hospital days of increased supplemental oxygen therapy, ICU admission, ICU days stay, Adverse events, Serious adverse events
Subramanian et al. [21]	1998	RCT	42	10 or 15 mg/kg palivizumab	0.9% saline	Safety, tolerance, immunogenicity, and pharmacokinetics of repeat intravenous doses of palivizumab
IMpact-RSV Study Group [19]	1998	RCT	1502	Palivizumab	Placebo	Safety and efficacy of prophylaxis with palivizumab in reducing the incidence of hospitalization

*Hospitalization for RSV Infection* 

Four studies reported the efficacy of palivizumab immunoprophylaxis against hospitalization for RSV infection. The analysis estimate showed a significant difference between palivizumab and placebo regarding the hospitalization for RSV infection, favoring palivizumab over the placebo (RR=0.51, 95% CI: 0.4 to 0.65; P<0.00001). No heterogeneity was detected between the studies as the pooled studies were homogenous (I2=0%, P=0.64).

All-Cause Mortality

**Figure 3:**
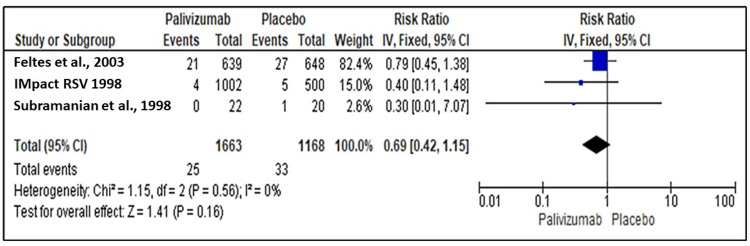
Forest plot showing the efficacy of palivizumab therapy regarding all-cause mortality Feltes et al. (2003) [18], IMpact RSV Study Group (1998) [19], Subramanian et al. (1998) [20]

Three studies [18, 19, 20] reported the efficacy of palivizumab and placebo for immunoprophylaxis against RSV regarding all-cause mortality. The analysis estimate showed no significant difference between palivizumab and placebo regarding all-cause mortality (RR=0.69, 95% CI: 0.42 to 1.15, P=0.16). No heterogeneity was detected between the studies as the pooled studies were homogenous (I2=0%, P=0.56) (Figure 3).

Lower Respiratory Tract Infection (LRTI)

**Figure 4:**
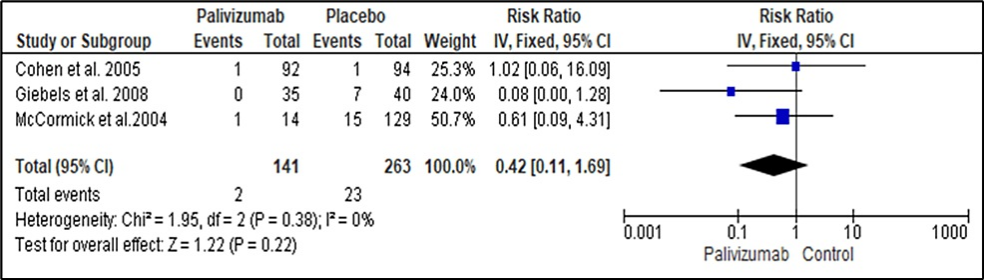
Forest plot showing the efficacy of palivizumab prophylaxis regarding the LRTI LRTI: lower respiratory tract infection Cohen et al. (2005) [27], Giebels et al. (2008) [22], McCormick & Southern (2007) [20]

Three studies reported the efficacy of palivizumab and placebo for Immunoprophylaxis against RSV regarding the incidence of LRTIs [20,25,27]. The analysis estimate showed no significant difference between palivizumab and placebo regarding the incidence of LRTIs (RR=0.42, 95% CI: 0.11 to 1.69; P=0.22). No heterogeneity was detected between the studies as the pooled studies were homogenous (I2=0%, P=0.22) (Figure 4).

Admission to ICU

**Figure 5:**
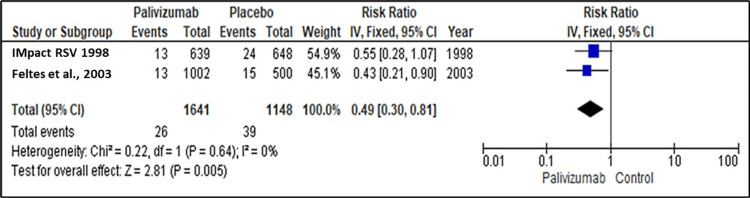
Forest plot showing the efficacy of palivizumab prophylaxis regarding ICU admission ICU: intensive care unit Feltes et al. (2003) [18], IMpact-RSV Study Group (1998) [19]

Two studies reported the efficacy of palivizumab and placebo for Immunoprophylaxis against RSV regarding ICU admission [18,19]. The pooled analysis showed a significant difference between palivizumab and placebo regarding the ICU admission after Immunoprophylaxis, favoring palivizumab over the placebo (RR=0.49, 95% CI: 0.30 to 0.81; P=0.005). No heterogeneity was detected between the studies as the pooled studies were homogenous (I2=0%, P=0.64) (Figure 5).

Mechanical Ventilation for RSV Infection

**Figure 6:**
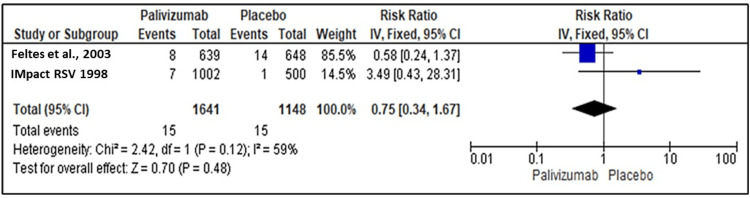
Forest plot showing the efficacy of palivizumab prophylaxis regarding mechanical ventilation for RSV infection RSV: respiratory syncytial virus Feltes et al. (2003) [18], IMpact-RSV Study Group (1998) [19]

Two studies reported the efficacy of palivizumab and placebo for Immunoprophylaxis against RSV regarding the mechanical ventilation for RSV infection. Palivizumab has no favorable effect in terms of mechanical ventilation reduction (RR=0.75; 95% CI {0.34, 1.67}; P=0.48). The pooled studies were heterogeneous (I2=59%, P=0.12), and the heterogeneity could not be resolved due to the limited number of included studies (Figure 6).

Number of Children Reporting Related Adverse Events

**Figure 7:**
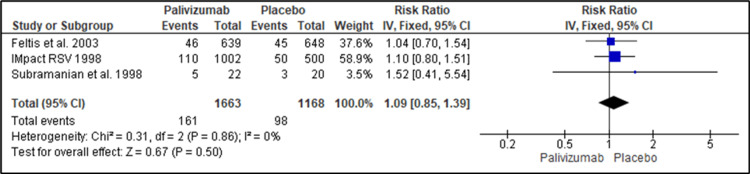
Forest plot showing the efficacy of palivizumab prophylaxis regarding the number of adverse events Feltes et al. (2003) [18], IMpact-RSV Study Group (1998) [19], Subramanian et al. (1998) [21]

Three studies reported the efficacy of palivizumab and placebo for immunoprophylaxis against RSV regarding the number of children reporting related adverse events. Palivizumab showed no favorable effect regarding related adverse events (AE) reduction (RR=1.09; 95%CI {0.85, 1.39}; P=0.5). No heterogeneity was detected between the pooled studies. The pooled studies were homogenous (I2=0%, P=0.86) (Figure 7).

Discussion

This systematic review and meta-analysis aimed to evaluate the efficacy and safety of palivizumab immunoprophylaxis for RSV in high-risk infants and children. The results demonstrate that palivizumab significantly reduces RSV hospitalization rates compared to placebo or no prophylaxis. The pooled analysis showed a 51% relative reduction in RSV hospitalizations with palivizumab prophylaxis. This finding is consistent with a previous systematic review that reported that monthly intramuscular injections of palivizumab during RSV season substantially protect against severe RSV disease requiring hospital admission in high-risk children [28].

The populations benefiting most from palivizumab include premature infants, infants with chronic lung disease or congenital heart disease, and young children with immunodeficiencies [29]. Palivizumab provides passive immunity by neutralizing the RSV F fusion protein to prevent viral entry into host cells. The standard dose of 15 mg/kg per month during RSV season aims to maintain protective serum concentrations above 40 μg/ml [30]. The significant reduction in RSV hospitalizations demonstrated in this meta-analysis supports current guidelines recommending palivizumab prophylaxis in high-risk pediatric groups [31].

However, this meta-analysis found no significant differences between palivizumab and placebo for other important clinical outcomes like all-cause mortality, LRTIs, number of adverse events, and need for mechanical ventilation. The wide confidence intervals suggest uncertainty in the effect estimates for these outcomes. Previous systematic reviews also did not find significant benefits of palivizumab on mortality or LRTIs [28,32]. The lack of risk reduction for mechanical ventilation and LRTIs indicates that palivizumab may not substantially impact RSV disease severity in hospitalized patients or the infant has another infectious agent that exacerbates the condition. 

Notably, palivizumab significantly decreased ICU admission rates compared to placebo, with a 49% relative reduction based on two RCTs. Preventing ICU admissions could greatly reduce healthcare costs and morbidity associated with severe RSV disease. However, only two small RCTs reported this outcome, so more data is needed to confirm this finding before making definitive conclusions about the effects of palivizumab prophylaxis on ICU admissions.

In a previous Cochrane systematic review, Robinson et al. (2016) [33] included only one trial of Cohen et al. (2005) [27], a double-blind, placebo-controlled trial conducted in 186 children with CF across 40 centers in the USA. They found a non-significant difference between palivizumab and placebo regarding the need for hospitalization (RR= 1.02, 95% CI: 0.06 to 16.09) or need for oxygen therapy (RR= 3.06, 95% CI: 0.13 to 74.27). After 12 months of follow-up, both groups did not record any single mortality event. They did not assess the length of hospitalization or ICU admission. Based on these findings, Robinson et al. (2016) concluded that it was impossible to draw firm conclusions on the safety and tolerability of RSV prophylaxis with palivizumab in infants with cystic fibrosis [33].

Another systematic review by Hu and Robinson (2010) that included two RCTs and five case reports and case series, showed that the RCTs were not powered to look at clinical outcomes, and concluded that palivizumab would need to be tested in larger RCTs before it could be recommended as an RSV treatment in any clinical setting [34].

Wong et al. (2018) [35] conducted a systematic review of 39 studies to identify the effect of adherence to palivizumab on its outcomes in patients with RSV. They found that the adherence rate was very high (>90%) in infants with RSV, as reported by RCTs, while in cohort studies, it ranged between 94% and 100%. However, out of the included studies, only seven have investigated adherence's impact on hospitalization rates. Their findings indicated that an adequate dosage of palivizumab with strict adherence to the medication positively impacted the hospitalization rates [32].

Mac et al. (2019) performed a systematic review of the cost-effectiveness of palivizumab in children with RSV [36]. Results from 28 worldwide economic evaluations showed that in prematurely born infants, infants with respiratory illness, and infants from rural areas, palivizumab was found to be cost-effective as an RSV prophylactic. These findings will support the utilization of such medication in primary and private healthcare centers under the guidance of healthcare authorities.

This meta-analysis has several strengths, including the comprehensive literature search of multiple databases and the inclusion of RCTs and observational studies. The limitations are the small number of RCTs in pooled analyses for some clinical outcomes and the high heterogeneity for the mechanical ventilation outcome. Only four RCTs were included in the meta-analysis, and they varied substantially in design, patient population, dosage regimens, and outcome definitions. Many of the observational studies were retrospective cohort studies with inherent biases. Finally, further well-designed, long-term studies are required to investigate the role of Nirsevimab in patients with RSV. Moreover, the utility of newly discovered adult vaccines in infants and children. 

## Conclusions

In conclusion, this systematic review and meta-analysis provide high-quality evidence that palivizumab immunoprophylaxis substantially reduces respiratory syncytial virus (RSV) hospitalization rates but may not impact other clinical outcomes like mortality and disease severity in high-risk infants and children. The use of palivizumab should be considered in combination with its high costs and potential adverse effects like injection site reactions. Further research is warranted on the efficacy of palivizumab prophylaxis in reducing ICU admissions, mechanical ventilation, and long-term respiratory morbidity. Cost-effectiveness studies are also needed to guide evidence-based recommendations on the appropriate use of palivizumab for RSV immunoprophylaxis in pediatric populations.
